# Ohio Dental College

**Published:** 1877-04

**Authors:** 


### Editorial

### OHIO DENTAL COLLEGE.

At the recent meeting of the Mississippi Valley Dental Society, the following resolution was unanimously adopted:

*Resolved:* That a committee of three be appointed to obtain contributions for the purpose of making some changes and improvements in the Ohio Dental College building, and also for providing some additional appliances and apparatus for illustration in teaching, and also to increase the library.

D. W. Roudebush,
Covington, Ky.
J. K. Jameson,
Connersville, Ind.
Jas. Leslie, Treasurer,
Cincinnati, O.

This movement on the part of this society will be very gratifying to the friends of the college. There is real need in the directions indicated in the resolution, or at least the legitimate work of the institution can be better done if these wants are supplied.

The college is owned by the profession, and should be supplied with every thing required to make its work most efficient. Every stockholder and every friend of the college should have a pride in this matter. The college should want for nothing that would increase its efficiency for good. Every graduate from it should feel and manifest in a substantial way, to the amount of his ability, his interest in, and appreciation of his *alma mater*.

Now we have a suggestion, and we trust that it will be received kindly and acted upon.

Let every stockholder, alumnus and friend of the college, at once forward to the treasurer of this commitee, some contribution, such an amount as you may choose, anywhere from one to fifty dollars.

Let not the committee be put to the trouble of asking any one for such contributions, but let them be sent in.

At the meeting quite a number came forward and promptly made their contributions; now let all do likewise.

